# A technology-assisted life of recovery from psychosis

**DOI:** 10.1038/s41537-019-0083-y

**Published:** 2019-09-18

**Authors:** Dror Ben-Zeev, Benjamin Buck, Sarah Kopelovich, Suzanne Meller

**Affiliations:** Department of Psychiatry and Behavioral Sciences, University of Washington School of Medicine, Seattle, WA USA

**Keywords:** Psychiatric disorders, Psychosis

## Abstract

Developments in digital health technologies have the potential to expedite and strengthen the path towards recovery for people with psychosis. This perspective piece provides a snapshot of how a range of digital technologies can be deployed to support a young adult’s efforts to cope with schizophrenia-spectrum illness. In conjunction with a day in the life of this individual, we provide examples of innovations in digital health research designed for this clinical population, as well as brief summaries of the evidence supporting the usability, feasibility, or effectiveness of each approach. From early detection to ongoing symptom management and vocational rehabilitation, this day-in-the-life vignette provides an overview of the ways in which digital health innovations could be used in concert to augment, scaffold, and enhance schizophrenia-spectrum illness management and recovery.

## Introduction

Imagine a future in which the exciting digital health and computational psychiatry breakthroughs we are seeing today lead to the development of effective technology-assisted illness detection, monitoring, and treatment-support tools of tomorrow.^1–3^ Public-private partnerships, rapid commercialization models, and new regulatory frameworks will create opportunities to make these instruments widely available and affordable.^4^ “Digital natives”^5^—individuals who grew up in the internet and smartphone era—will be fully capable of using familiar technologies for personal health support.^6,7^ Our scientific understanding of psychopathology will progress to a point that we may no longer view psychosis as a fixed and hopeless state but as a continuum of experience that varies both across people and within the same individual over time.^8,9^ When these conditions converge, a range of technology-based and technology-assisted mental health resources will provide individuals with psychosis more opportunities to actualize their potential for recovery. In what follows, we describe what the daily life of a young adult in recovery from psychosis may look like in what we believe to be the not-too-distant future.

Annie is a 21-year-old woman in her junior year of college. She works part-time at a flower shop in her neighborhood, owns a 4-month-old energetic puppy that she adopted from the local animal shelter, and is in a committed relationship with her boyfriend of two years. Annie began to experience depressed mood and derogatory auditory hallucinations just over a year ago. Feeling anxious about her wellbeing, she Googled “where do voices come from,” “hearing whispers,” and “do I have schizophrenia”. *[The Pew Research Center found that one in three American adults have gone online to try to understand a health condition*.^10^ *Google AdWords have been employed as part of a digital outreach campaign*, *resulting in over four thousand click-throughs to psychosis-specific psychoeducation*, *psychosis self-screening*, *and encouragement to reach out to local early psychosis clinical services*.^11^*]* She completed an online self-report mental health screener that alerted her to a need for additional assessment. *[An online-administered screener for psychosis has demonstrated positive predictive value that exceeded that of clinician-administered interview in estimating psychosis conversion*.^12^ *Risk calculators incorporating demographics*, *family history and other clinical and functional indicators have demonstrated promise and are similarly available online*.^13^*]* As a part of this follow-up assessment, she recorded several prompted speech samples using her laptop, *[Natural Language Processing (NLP) systems with automated machine learning*^14,15^ *have demonstrated promise in strengthening predictions of psychosis risk*.^16^ *One approach deployed in a high-risk sample demonstrated cross-validated accuracy predicting psychosis onset*.^17^*]* and received neuroimaging at a nearby testing center. *[Machine-learning analytics of functional imaging data generated correct identification of schizophrenia in a majority of newly diagnosed individuals*. *It could further predict which patients would respond to treatment with antipsychotic medication*.^18^*]*

The early psychosis specialty clinic to which Annie was referred was nearly two hours’ drive by car. Her parents took the day off of work to drive Annie to her appointment. The intake specialist reviewed her online assessments and NLP results prior to her intake appointment, which permitted him to focus more on discussing her and her parents’ concerns, strengths, resources, and aspirations through the use of a web-based clinician support tool aimed to enhance shared decision-making practices. *[Computerized clinician support tools may enhance delivery of best- and evidence-based practices*.^19^*]* Annie was also able to receive a tour of the clinic, where she learned that she would be able to access her care team virtually, was introduced to her personalized digital health dashboard, received a brief orientation to her personalized menu of digital health tools, and received her first telehealth appointment for the following day. Annie and her parents were relieved that she would not need to travel back to the clinic for this appointment.^20,21^

Like most of the adult population, including people with severe psychiatric conditions, Annie owns a mobile phone.^22,23^ In the 12 months since she established care, this personal device has become her primary mental health support system. Every morning Annie receives a tailored text message that reminds her to take her antipsychotic medications. *[Multiple approaches using text messages to remind users about their medications have been deployed and appear feasible and highly usable among people with serious mental illness*.^24,25^*]* While she is eating breakfast Annie receives an instant message from her community-based case manager who reminds her that her driving exam is next week and that her rent is due. *[A study of “hovering” treatment via text messages demonstrated that participants with serious mental illness respond to the majority of clinical messages sent by their community-based mobile interventionist and most find this intervention useful*.^26^*]* Computer-based cognitive remediation training has improved her ability to focus and she was able to study the Department of Motor Vehicle’s driver manual without difficulty. *[Meta-analyses suggest that cognitive remediation interventions demonstrate positive medium effects for cognitive performance and functioning and small effects for symptoms*.^27,28^*]*

Annie has a work shift before her afternoon classes. She found her job at the flower shop with the aid of a community-based supported employment specialist who also introduced her to a software program that helped her develop the skills and confidence necessary to pass the job interview successfully. *[Virtual reality job interview training has been shown to improve job interview role-play performance and real-world job attainment in a randomized controlled trial*.^29^*]* The same specialist provides her with on-the-job follow-along support regularly via a supported employment app she uses to cope with job-related challenges and stress. *[A feasibility study of a mobile application designed to support the employment of individuals with serious mental illness in real time found that the majority of users found the app to be user-friendly and helpful in providing on-the-job supports*.^30^*]* She completes her shift and heads over to campus for class. Before entering the lecture hall, she experiences heightened anxiety coupled with auditory hallucinations that berate her, saying that she is not smart enough to understand this subject. This is not an uncommon experience for Annie. Fortunately, she has adopted effective technology-supported coping strategies; she finds a private spot, takes out her smartphone, and activates her illness self-management app. Annie views several videos that guide her through a sequence of relaxation strategies and cognitive restructuring techniques focused on dysfunctional beliefs linked with the experience of hearing voices. *[A smartphone self-management intervention called FOCUS has been shown to be usable*,^31^*feasible*,^32^ *engaging*, *and clinically effective*.^33^*]* In just a few minutes she feels calmer and is able to find her seat and fully engage in the class. She activates her smartphone to audio record today’s lecture.

Picking up her phone after class, Annie notices that she has an encouraging text message from her mother. Over the last few months, her mom has been participating in an online group of family members of young adults with psychosis. Since she joined, Annie has noticed that her parents are less anxious when Annie talks about her symptoms. As a result, Annie has been more open and their relationship has improved. *[Proof-of-concept studies have demonstrated that online psychoeducation for individuals with schizophrenia and their family members is feasible and acceptable*.^34^ *A web-based family and client psychoeducation intervention has demonstrated significant improvements in positive symptoms and knowledge about schizophrenia*.^35^*]* Leaving class, she checks the likelihood of traffic on her commute on her phone. Because walking through crowds increases her anxiety and paranoia, she had been previously intentionally avoiding crowds on her commute. However, today, she decides to take the quickest (but also busiest) way home. Over the past few weeks, she has been challenging her beliefs about the dangerousness of crowds with a virtual reality (VR) self-training tool, and she decides today’s the day she’s going to give this route a shot. *[Virtual reality cognitive therapy for persecutory ideation has been shown to reduce delusional belief conviction and associated distress*.^36,37^*]*

When Annie returns home from class, she grabs a handful of pretzels and a soda from the fridge. She has an online session scheduled with her therapist just before dinner and wants to snack before the call. *[Individuals with psychosis use and are satisfied with clinical services delivered via two-way videoconferencing*.^38^*]* As she drinks her soda, she fills out a brief outcome questionnaire on her phone that will be reviewed by her clinician ahead of their session. In the free-text window, she writes that she walked through the busiest parts of campus today and it went very well. *[Mobile apps deploying remote outcome assessments appear feasible and acceptable in specialty early psychosis settings*, *and generate ratings that are similar to gold-standard clinical tools*.^39^ *Such systems provide opportunities for monitoring and personally tailored care*.*]* She is excited to share the news about her successful exposure with her clinician later that evening. During the session, her therapist displays to Annie her symptom assessment scores over the last four months of treatment. Annie notices that her anxiety has diminished since engaging in exposure treatment, and her voices have also become less intense and malevolent.^40^ Annie and her therapist reflect on this pattern and discuss the coping strategies that seemed to have helped the most. Guided by her therapist, Annie selects coping strategies that she will employ to help self-regulate distress during her upcoming driver’s test. Next, Annie and her therapist continue to treat her voices-related distress. Annie’s therapist presents her voice avatar on the screen, so that Annie can become more comfortable tolerating the voices and practice rational responses when the voice content is derogatory. *[A computerized treatment has shown therapeutic effects on auditory verbal hallucination severity by encouraging individuals to engage in a dialogue with digital representations of their voices*.^41^ *This work has been extended in a randomized trial*.^42^ *More immersive*, *virtual reality platforms have also demonstrated positive effects*.^43^*]*

Because Annie’s therapist is still in a Cognitive Behavioral Therapy for Psychosis (CBTp) training program, Annie has consented to have their sessions recorded and reviewed by a CBTp-trained supervisor. When the pair log off, the session is uploaded to a remote server so that her therapist can receive feedback from her supervisor.^44^ In addition to personalized, specific, and prompt feedback on her competence and adherence, Annie’s therapist can virtually access continual education, support, and telementoring via an online portal. *[Web-based training*, *followed by peer-led consultation with previously-trained providers appears to be a viable way to enhance knowledge acquisition and competency*.^45^ *Telementoring among community mental health clinicians learning CBTp enhances confidence and affects their approach with patients*.^46^*]*

Meanwhile, after logging off from her therapy session, Annie updates her online early psychosis community with a post to the group feed. She had been sharing her experiences with peers online and several others had encouraged her to try the VR program. She is excited to let them know that it was helpful! *[An online social community for young adults with early psychosis has been examined in a feasibility study*, *revealing high system usage over a 3-week period*.^47^*]* Annie closes her laptop and takes a look at her phone. She sees a calendar reminder that she’s scheduled to meet with friends for dinner and a movie.

On the way to the restaurant, she passes several liquor stores. In her pocket, her phone generates several new notifications reminding her that alcohol can exacerbate her symptoms. She remembers that a few years ago, when she was starting college and struggling with significant depression, she was drinking heavily. *[A mobile app supports the recovery of individuals with substance abuse concerns by providing them with information and support as well as a monitoring feature that prompts app users when they are near high-risk locations as detected by GPS*.^48^*]* Annie has a great time at dinner and at the movie with her friends, and after the movie ends, like so many other Americans, she uses her rideshare app to request a ride home. While she is waiting for her car to arrive she kills time by watching today’s vlog update by an individual with lived experience she has been following who posts his recovery testimonials on YouTube. *[Survey studies suggest that young adults with early psychosis often turn to YouTube as a primary source for information and virtual community support related to mental illness*.^49,50^*]* She chuckles as she reads some of the comments and is reminded how so many like her with a serious mental illness not only cope, but also thrive.

## Discussion

The peer-reviewed literature is now replete with descriptions of novel digital health approaches for help-seeking individuals with psychosis. The evidence suggests that individuals with psychosis use electronic devices, digital applications, and social media resources in a manner that is comparable to those without psychosis. Annie’s story paints a picture of how a young digital native may use a variety of technologies to improve their quality of life, extend symptom management into their day-to-day activities, and facilitate wellness. Many of the tools we reference in the vignette are in early phases of development (e.g., proof of concept, feasibility), and more rigorous testing is needed to ascertain for whom and in what contexts they are most suitable. Future developments will not only determine the specific components of these interventions, but how best to integrate them with existing mental health services.

The ubiquity and increasing utility of technology in our everyday lives may concern those who fear the overreliance on technology, privacy breaches, or that digital health technologies will replace human-delivered assessment and treatment. These concerns must be balanced with the potential for technology to enhance care and healthy practices, whether employed independently or with the assistance of a care team. The coordinated specialty care (CSC) model has transformed the standard of in-person care for first-episode psychosis, emphasizing a team-based, multi-element approach to address the complex and varied needs of this population.^51^ Still, significant barriers, including cost, workforce development, and community involvement remain in expanding access to these interventions.^52^ As demonstrated in Annie’s narrative, technology can be additive to human-delivered, evidence-based treatment. These technologies are consistent with the multi-component emphasis of CSC, as they connect her with others to create a team of community case managers, employment specialists, therapists, and people with lived experience that can help her succeed.

In many respects, this “day-in-the-life” is an ideal. In a typical day there are many opportunities for an individual experiencing psychosis to either engage in protective or risky behaviors. Over time, these coping skills have cumulative effects and important implications for mental health, functioning, self-determination, and quality of life. Emerging technologies may serve as the key scaffold to implementing effective practices in a scalable, personalized, and sustainable manner. While we can imagine this future today, the developments of tomorrow will determine whether we realize this vision.

## Data Availability

No original data were used in the preparation of this article.
